# Prediction of the mechanism for the combination of diallyl trisulfide and cisplatin against gastric cancer: a network pharmacology study and pharmacological evaluation

**DOI:** 10.3389/fphar.2023.1269895

**Published:** 2023-10-30

**Authors:** Huaiyou Lv, Xiumei Jia, Huatian Yang, Xiaosong Zhu, Zhongxi Zhao, Xiaoyan Jiang

**Affiliations:** ^1^ Department of Pharmacy, Qilu Hospital of Shandong University, Jinan, Shandong, China; ^2^ Department of Pharmaceutics, Shandong University, Jinan, Shandong, China; ^3^ Department of Pharmacy, Yantai Yuhuangding Hospital Affiliated to Qingdao University, Yantai, Shandong, China; ^4^ Department of Infection Management, Linyi People’s Hospital, Linyi, Shandong, China

**Keywords:** gastric cancer, diallyl trisulfide, cisplatin, network pharmacology, MAPK/STAT3/PKC-δ, endoplasmic reticulum stress

## Abstract

**Background:** In this research, we aimed to explore the efficacy of diallyl trisulfide (DATS) combined with cisplatin (DDP) for gastric cancer treatment and its underlying mechanism based on network pharmacology.

**Methods:** First, the pharmacological mechanism by which DATS combined with DDP acts against gastric cancer was predicted using network pharmacology. The TTD, GeneCards, and OMIM databases were used to extract drug and disease targets. The David Bioinformatics Resources 6.8 database was used to conduct GO and KEGG analyses. We investigated the efficacy of DATS combined with DDP against gastric cancer in SGC7901 cells and a xenograft model. Furthermore, the specific mechanism of DATS combined with DDP, inferred by network pharmacology, was identified by Western blotting and immunohistochemistry.

**Results:** The combination of DDP and DATS significantly increased cytotoxicity and cell apoptosis compared to the DATS or DDP treatment group *in vitro*. In addition, continuous intraperitoneal injection of DATS markedly improved the tumor inhibitory effect of DDP in the SGC-7901 tumor-bearing mouse model. Furthermore, network pharmacology and experimental validation studies revealed that the combination of DATS and DDP synergistically enhanced antitumor activity by regulating endoplasmic reticulum stress and inhibiting STAT3/PKC-δ and MAPK signaling pathways.

**Conclusion:** Our study showed that the combination of DATS and DDP could exert outstanding therapeutic effects in gastric cancer. Moreover, network pharmacology coupled with experimental validation revealed the molecular mechanisms of combination therapy for gastric cancer. This study offers a new adjuvant strategy based on DATS and DDP for the treatment of gastric cancer.

## 1 Introduction

Gastric cancer (GC) is the fifth most confirmed malignant tumor worldwide, with an annual increase of more than 1 million cases. Owing to its advanced stage at diagnosis, it has become the third leading cause of cancer-related deaths (Sung et al., 2021). Continuous chemotherapy based on first-line platinum and fluoropyrimidine has been used in the treatment of advanced gastric cancer. Cis-diaminedichloroplatinum II (DDP, cisplatin) is a widely used first-line chemotherapy drug and the main treatment strategy for patients with advanced GC, and has been shown to effectively improve the survival rate of patients with advanced GC (Shah, 2015; Sasaki et al., 2017). However, DDP exhibits drug resistance and significant organ toxicity, such as nephrotoxicity, ototoxicity, hepatotoxicity, and gastrointestinal toxicities (Ghosh, 2019). After several cycles of chemotherapy with DDP, approximately one-third of the patients developed renal dysfunction (Merouani et al., 1996), and half of the patients showed acquired drug resistance. Therefore, various new adjuvant approaches are urgently needed to protect normal organs from damage and enhance the therapeutic effect of DDP on gastric cancer cells or tumors, which could provide new ideas to improve DDP-based cancer treatment.

Garlic (*Allium sativum*) is an ancient cultivated plant that possesses the dual-purpose characteristics of food and medicine. Epidemiological and research studies have demonstrated that many active components of garlic have preventive and suppressive effects on various types of tumors (Iciek et al., 2009; Tsubura et al., 2011; Jin et al., 2013; Yan et al., 2014). Recent research has shown that basic treatment with *H. pylori* (H.pylori) and garlic supplementation for 7 years significantly decreased the risk of death from gastric cancer (Li et al., 2019). Garlic extract products possess activity against *Helicobacter pylori* and gastric inflammation, which promotes its potential role in the treatment of peptic ulcer diseases (Sivam, 2001). Moreover, garlic powder, aged garlic extract, and some organosulfur compounds (s-allylcysteine, diallyl disulfide, and diallyl sulfide) have been reported to exhibit antioxidant effects that can ameliorate the nephrotoxic effects of DDP (Dwivedi et al., 1996; Chiarandini Fiore et al., 2008; Razo-Rodriguez et al., 2008; Gomez-Sierra et al., 2014; Nasr and Saleh, 2014). Diallyl trisulfide (DATS), a major bioactive compound in garlic, has multiple health benefits such as anticancer, anti-inflammatory, and immunomodulatory effects. Due to the existence of a triple sulfur bond (-S-S-S-) structure, DATS was reported to be more effective than single and double sulfur-containing compounds in antitumor activity and chemical detoxification defense functions (Tsai et al., 2005; Lai et al., 2013). In addition, DATS has been proven to have a variety of active metabolites *in vivo* in drug metabolism and pharmacokinetics (DMPK) studies, which might offer better protective and therapeutic effects depending on multiple target strategies (Gao et al., 2013). Nevertheless, existing research on garlic organic sulfur compounds has focused on their anti-tumor and anti-oxidation properties. Based on the characteristics of garlic ingredients in dietary therapy, it is worth exploring in-depth in the joint treatment strategy.

Network pharmacology, as a new scientific approach for drug combinatorial research, has the characteristics of constructing “drug-gene-target-disease” networks based on biological properties, analyzing the linkages between drugs, proteins/genes, and diseases, and predicting the mechanism of action of small molecule drugs on diseases (Nogales et al., 2022). Network pharmacology offers brilliant prospects for multiple disease treatments rather than targeting a single disease target. Given its systematic and comprehensive characteristics, more and more research on the active substances in herbal medicines and their mechanisms of action on disease targets were based on network pharmacology (Li et al., 2022).

In this study, we attempted to apply network pharmacology to predict the mechanism of action of DATS combined with DDP against gastric cancer and then validated its protective effect as well as the possible mechanism through pharmacological experiments *in vitro* and *in vivo*. This research revealed the effects and molecular mechanism of the combination of DATS and DDP, which could provide novel ideas for chemotherapy of gastric cancer.

## 2 Materials and methods

### 2.1 Drugs and chemicals

DATS (98% purity) was availible from AIKE Biotechnology (Chengdu, China). DDP was obtained from Qilu Pharmaceutical (Jinan, China). Antibodies against Akt, phospho-Akt, phospho-ERK1/2, ERK1/2, phospho-p38, p38, phospho-JNK, JNK, and PKC-δ were obtained from Cell Signaling Technology (Danvers, MA, United States). Antibodies against XBP1, IRE1α, Calnexin and GAPDH were purchased from Beyotime Biotechnology (Shanghai, China). The apoptosis detection kit, cell mitochondrial membrane potential assay kit (TMRE), Annexin V-FITC apoptosis detection kit, TUNEL staining kit, Cell Counting Kit-8 (CCK-8) and calcium ion fluorescent probes (Fluo 3-AM) were purchased from Servicebio Biotechnology (Wuhan, China). Transwell Chambers and Matrigel were obtained from Corning (New York, NY, United States).

### 2.2 Collection of gene symbols for gastric cancer and construction of protein-protein interaction (PPI) networks

First, the targets of DDP combined with DATS were identified by importing the chemical structures into the SwissTargetPrediction data library (http://www.swisstargetprediction.ch/). After standardizing the target names by the UniProt database (https://www.uniprot.org/), the underlying gene targets for gastric cancer were selected from the following three databases, GeneCards (https://www.genecards.org/), TTD (http://db.idrblab.net/ttd/), and OMIM databases (http://omim.org/). Next, the protein-protein interaction (PPI) network of DDP combined with DATS treatment in gastric cancer was obtained by uploading the selected intersecting genes of 3 databases into STRING 11.0 (http://stringdb.org/cgi/input.pl).

### 2.3 Definition of target pathway by building networks

The drug target network was constructed by Cytoscape 3.7.1 software to explore the molecular mechanism of DDP combined with DATS against GC. The potential pathways of DDP combined with DATS against GC were analyzed using gene ontology (GO) enrichment and KEGG pathway analyses using the David Bioinformatics Resources 6.8 database (https://david.ncifcrf.gov/).

### 2.4 Cell culture and cell proliferation

SGC-7901 cells were used as a gastric cancer model and were obtained from Cobioer Biotechnology (Beijing, China). SGC-7901 cells were cultured in RMPI-1640 medium with 10% fetal bovine serum (BioInd, Israel) at 37°C in a 5% CO_2_ incubator. The percentage inhibition of cell growth for 24 h by different concentrations of DATS and DDP was determined using the CCK-8 kit and following its instructions with 450 nm absorbance by a Microplate Reader (Safire2, TECAN, France). The combination index (CI) analysed by Calcusyn 2.1 software (Biosoft, Cambridge, United Kingdom) was used to reveal the interaction between DATS and DDP. CI < 1 indicated synergistic interactions (Chou, 2010).

### 2.5 Cell apoptosis

SGC-7901 cells were cultured in six-well plates and treated with the indicated doses of DATS, DDP, or their combination for 48 h. The cells were collected and stained with apoptosis detection kit according to the manufacturer’s instructions. To evaluate apoptosis, the cells were analyzed by flow cytometry (Beckman FC 500, United States of America) and calculated using FlowJo software.

### 2.6 Cell migration assay

The migration of SGC-7901 cells was examined by transwell cell migration and scratch assays. For transwell, the matrigel was diluted at a ratio of 1:8 with serum-free medium, and 40 μL diluted matrigel was spread on the upper layer of the chamber. SGC-7901 cells were adjusted to 5 × 10^5^ cells ml^−1^ using serum-free medium, then 100 μL cells and 100 μL drugs were added to the upper layer of the chamber, and 500 μL medium with 10% FBS was added to the lower layer of the chamber. After 48 h of incubation at 37°C, cells were fixed with 4% paraformaldehyde. After removing the cells in the upper compartment, the cells were stained with 0.1% crystal violet. The migrating cells were recorded under the microscope. Finally, photographs were taken using an inverted microscope. The number of cells migrating between the chambers was calculated using the ImageJ software. The scratch test was performed according to a standard protocol (Chou, 2010).

### 2.7 Calcium ion and mitochondrial membrane potential detections

SGC-7901 cells were plated on glass coverslips and incubated for 24 h. The cells were washed twice with PBS, fixed with 4% formaldehyde for 15 min, permeabilized with 0.2% TritonX-100 for 20 min, and blocked with 5% BSA for 0.5 h. After incubation with Fluo-3 AM and TMRE at 4°C for 1 h, the cells were washed thrice, followed by incubation with Hoechst for 5 min. Finally, images were obtained using an LSM900 confocal laser microscope (Carl Zeiss AG, Germany). The fluorescence intensity was quantified using flow cytometry.

### 2.8 Western blot

SGC-7901 cells were harvested and lysed in lysis buffer. Total protein was extracted and loaded into each well of 8%–10% SDS-PAGE gel and blotted onto PVDF membranes. The membranes were incubated with primary antibodies (1:1000 dilution) overnight at 4°C after blocking. After washing with TBST buffer three times, incubation with 1:5000 dilution of secondary antibodies linked to horseradish peroxidase (Bioss, China) were carried out for 2 h. Finally, the blots were visualized using ECL Plus reagent (Millipore Corp, Bedford, MA, United States). GAPDH was used to assess the amount of the reference protein in each lane. The relative protein levels were analyzed using AlphaView SA software.

### 2.9 *In vivo* tumor xenograft experiment

Female athymic nude mice (16–18 g) were purchased from the Institute of Laboratory Animal Sciences (Beijing, China). 5 × 10^6^ SGC-7901 cells in 100 µL PBS were injected subcutaneously into the armpit of the right forelimb of the mice. As the tumor volume (TV) reached approximately 100 mm^3^, four groups of mice (*n* = 6) were randomly divided and injected intraperitoneally with vehicle, DATS, DDP, and their combination: (i) vehicle (Control group), every day; (ii) DATS, 30 mg/kg every day; (iii) DDP, 4 mg/kg every 7 days; (iv) DATS+DDP, 4 mg/kg of DDP every 7 days, and 30 mg/kg of DATS every day. Tumor length (a), width (b), and body weight (BW) were recorded every 4 days, and TV was calculated as follows: TV = (a × b^2^)/2. After 32 days, the mice were sacrificed, and the main tissues were acquired for further assessment.

### 2.10 Histopathologic analysis

Tumor tissues were fixed in paraformaldehyde and embedded in paraffin. Sections were made and stained with hematoxylin and eosin to evaluate histopathological changes from three independent images using an Olympus microscope (Tokyo, Japan).

### 2.11 Immunohistochemistry and TUNEL assay

The immunohistochemical expression of calnexin, PKC-δ, XBP1, and p-JNK was determined. A series of xylene and ethanol aqueous solutions were used to dewax and rehydrate paraffin sections. After blocking, the sections were treated with microwaves to repair the antigen and expose antigenic determinants. After serum blocking, the paraffin sections were incubated with the primary antibody at a 1:100 dilution at 37°C in a humidified chamber for 2 h and then incubated with the diluted secondary antibody for 1 h. The specific binding antibodies in the sections were developed using DAB substrate and hematoxylin counterstain. While, according to the instructions from the TUNEL staining kit, DNA of tissue sections was also counterstained by DAPI. Images were acquired by an Olympus microscope (Tokyo, Japan).

### 2.12 Statistical analysis

Data were showed as mean ± SD and statistically evaluated by one-way ANOVA based on GraphPad Prism 6.0 statistical software. The significant differences between groups was evaluated by Unpaired *t*-test, with *p < 0.05* the criterion for statistical significance.

## 3 Results

### 3.1 Construction of PPI network of DDP and DATS against GC

**FIGURE 1:**
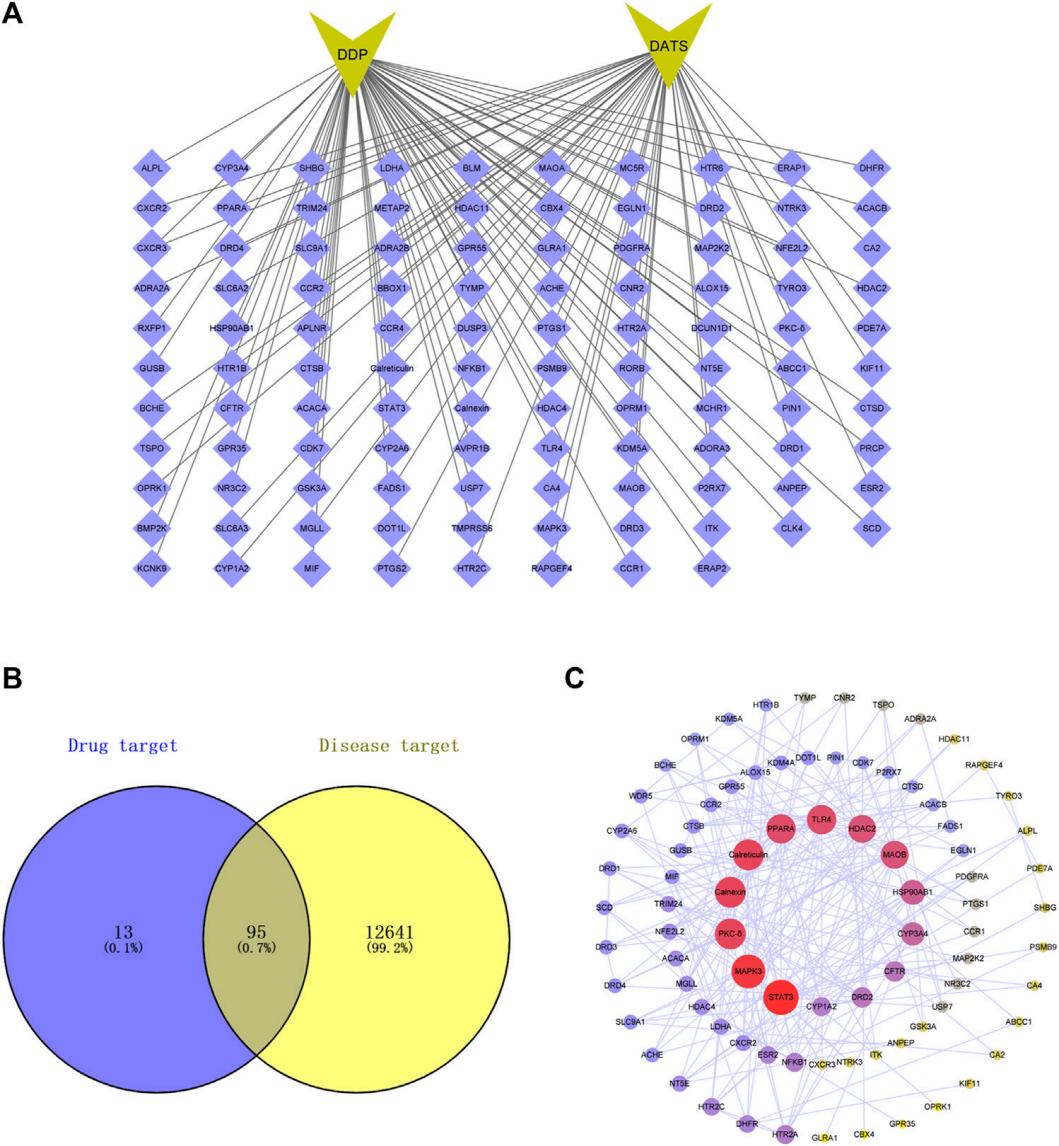
Protein-protein interaction (PPI) networks of potential targets for the DDP combined DATS treatment of GC. **(A)** Top 100 targets of DATS combined DDP in SwissTargetPrediction; **(B)** 95 targets of DATS combined DDP related to GC by Venn diagram; **(C)** PPI networks of potential targets for DATS combined DDP against GC.

As shown in Figure 1, the top 108 targets of DATS combined with DDP were selected from SwissTargetPrediction data (Figure 1A) while 12641 targets for GC were gathered from 3 databases. Consequently, 95 overlapping targets related to DATS, DDP, and GC were identified (Figure 1B). Protein intersections were then performed by mapping the targets of DATS combined with DDP on GC in the STRING database. Three topological features (betweenness centrality, degree and closeness centrality) focused on identification of the key targets in the network. A total of 45 points were identified as key targets in the network, including Calnexin, STAT3, PKC-δ and Calreticulin (Figure 1C).

### 3.2 KEGG and GO enrichment analysis

**FIGURE 2:**
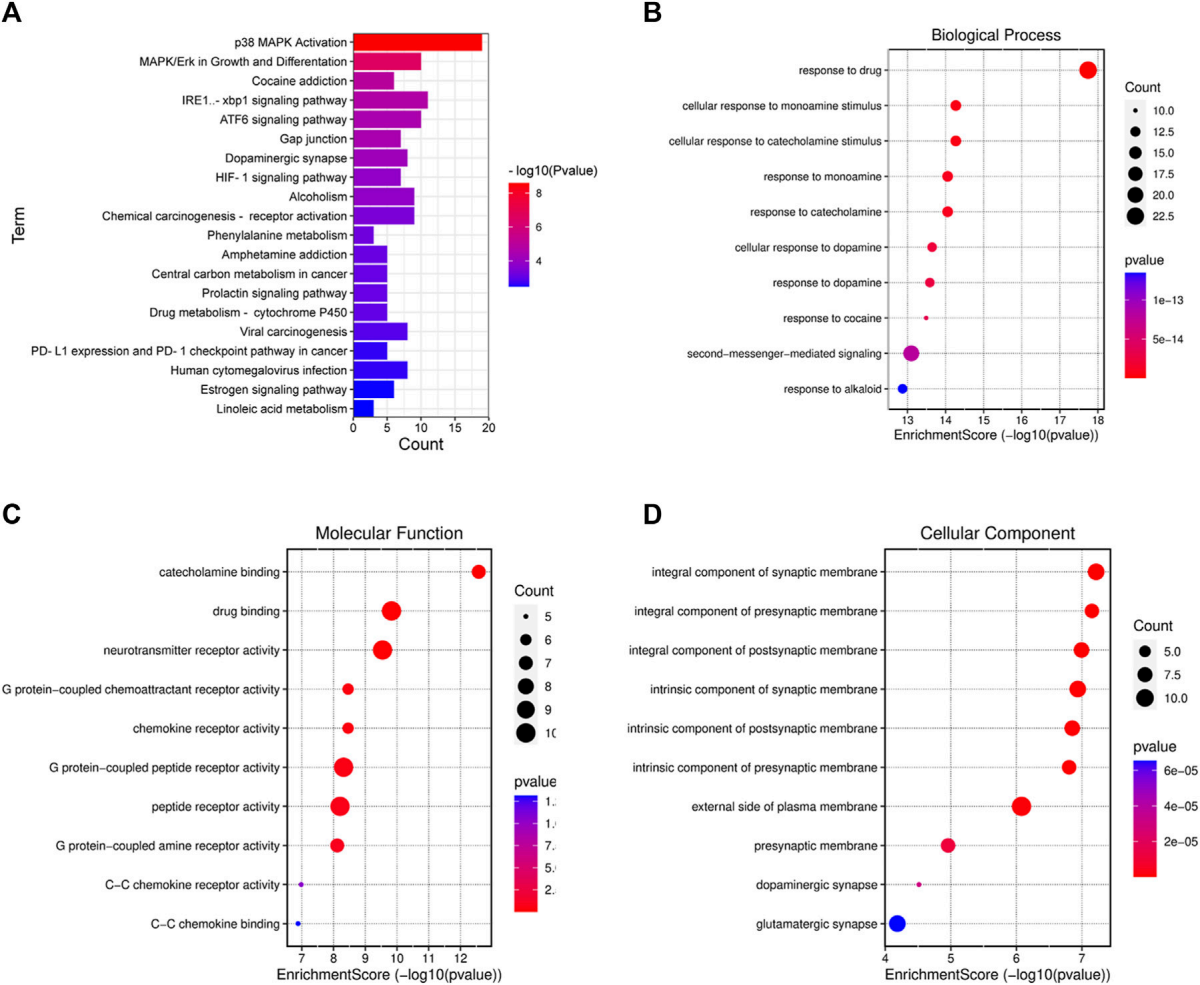
Diagrams of mechanistic analyses of **(A)** Pathways enrichment; **(B)** Biological process enrichment; **(C)** Molecular function enrichment; **(D)** Cellular component enrichment.

To confirm the biological properties of the relevant targets of DATS combined with DDP on GC in detail, KEGG and GO enrichment analysis was performed as shown in Figure 2. Through KEGG pathway enrichment, 83 related pathways were identified, of which the top twenty pathways are shown in Figure 2A.The P38/MAPK, IRE1/XBP1, and ATF6 signaling pathways are the three main pathways closely associated with proliferation and endoplasmic reticulum stress in GC.

GO analysis showed that 133 genes were selected among 2684 GO entries (*p* < 0.05), consisting of 2181 biological progress (BP), 215 cellular components (CC), and 288 molecular functions (MF). The top 10 entries of biological progress, cellular components, and molecular functions among the selected 133 genes are shown in Figures 2B–D, respectively. The potential targets among the top biological processes were primarily focused on the response to the drug and the cellular response to monoamine stimulus. In addition, the potential cellular targets were mainly focused on the integral components of the synaptic and presynaptic membranes. The major molecular functions are related to catecholamine and drug binding.

### 3.3 Effects of DATS and DDP on the proliferation and apoptosis of SGC-7901 cells

**FIGURE 3:**
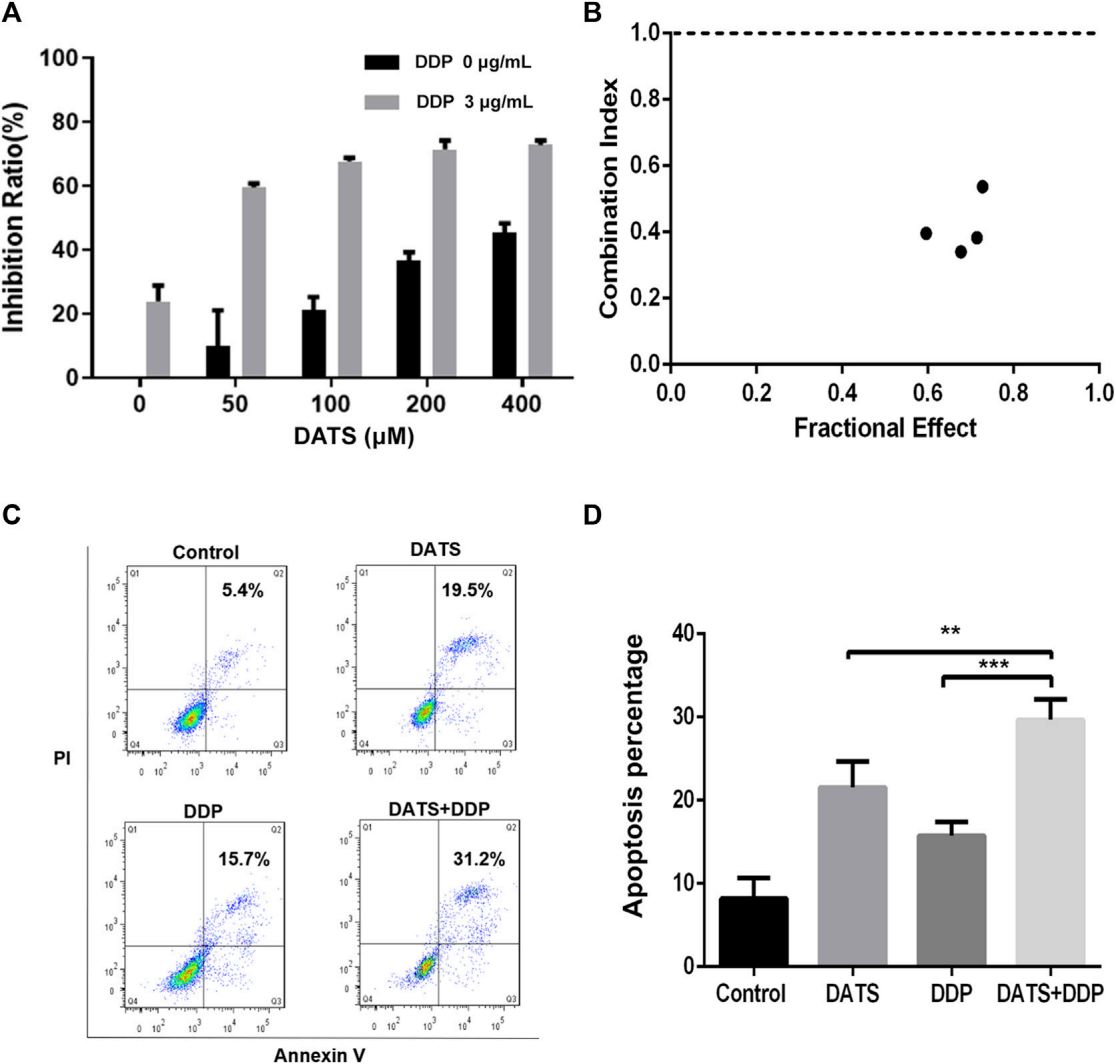
DATS synergistically enhanced the effects of cell growth inhibition and apoptosis of DDP in SGC-7901 cells. **(A)** The inhibition rate of SGC-7901 cells; **(B)** The CI values of DATS and DDP; **(C)** Apoptosis of SGC-7901 cells; **(D)** Apoptosis ratio of SGC-7901 cells after different treatments. Data are shown as mean ± SD (*n* = 3). Compared with the DATS+DDP group, ^*^ *p* < 0.05, ^**^ *p* < 0.01, ^***^ *p* < 0.001.

To determine the potential anticancer activity of DATS and DDP in combination, the CCK-8 assay was used to evaluate cell viability after treatment with DATS alone or in combination with DDP by CCK-8 assay. As shown in Figure 3A, DATS treatment (50–400 μM) resulted in dose-dependent growth inhibition of SGC-7901 cells, which increased from 16.20% ± 6.56% to 45.48% ± 6.91%. Nevertheless, when combined with 3 μg/mL DDP, the inhibition rate of DATS against SGC-7901 cells increased from 59.5% to 72.7%, which showed that DATS and DDP in combination significantly enhanced the cytotoxicity of SGC-7901 cells in comparison with DATS alone. In addition, CI values calculated using Comsyn software showed a strong synergy for the combination of DATS and DDP (CI < 1; Figure 3B). To explore the influence of the combination of DATS and DDP on apoptosis (Jiang et al., 2017), cell apoptosis was evaluated using flow cytometry. As shown in Figures 3C, D, compared to the two monotherapy groups, the combination treatment dramatically increased the apoptosis ratio in SGC-7901 cells. Apoptosis rate in the combination group was enhanced to 29.7% ± 2.4%, which was much higher than that in the two monotherapy groups (21.6% ± 2.0% for DATS and 13.9% ± 2.5% for DDP).

### 3.4 Effects of DATS and DDP on the metastasis of SGC-7901 cells

**FIGURE 4:**
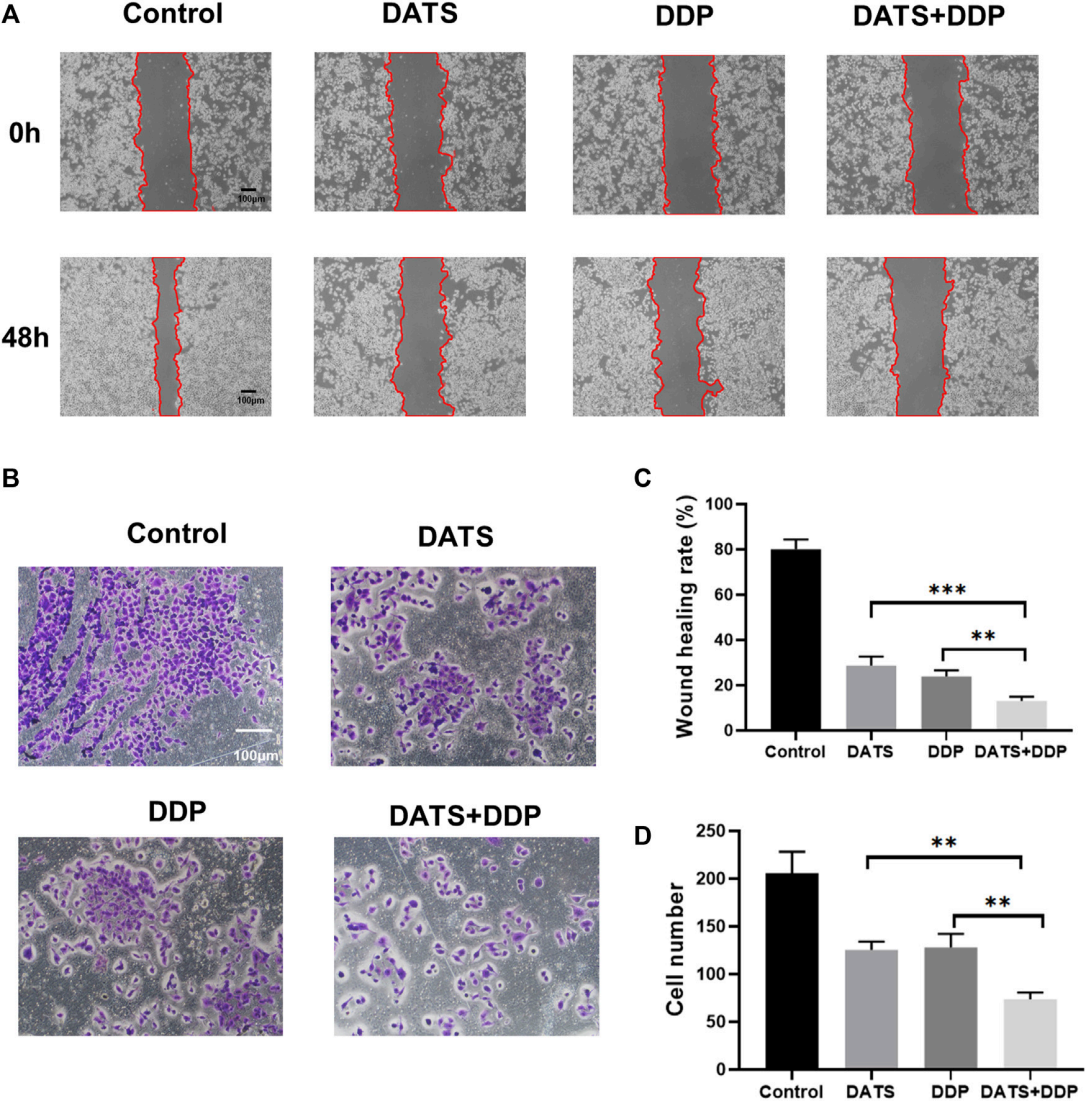
DATS synergistically enhanced the anti-migration of DDP in SGC-7901 cells. **(A)** The effects of DATS+DDP on the migration of SGC-7901 cells; **(B)** The effect of DATS+DDP on the Invasion ability of SGC-7901 cells. **(C)** Wound healing rate. **(D)** Cell number. Data are shown as the mean ± SD (*n* = 3). Compared to the DATS+DDP group, ^*^ *p* < 0.05, ^**^ *p* < 0.01, ^***^ *p* < 0.001.

To explore whether DATS and DDP affect GC metastasis, the effects of DATS and DDP in combination on cell invasion and migration were evaluated. Both the DATS and DDP groups exhibited the ability to inhibit cell migration compared with the control group. Interestingly, we observed that the inhibition of migration in the combination group was stronger than that of either DATS or DDP alone (^***^ *p* < 0.001, ^**^ *p* < 0.01, respectively) (Figures 4A, C). In addition, as can be seen in Figures 4B, D, compared with the control group, the number of cells invading the bottom of the vesicles was significantly reduced after DATS and DDP treatment, and this effect was remarkably enhanced in the co-treatment group of DATS and DDP as compared with the groups of single treatment.

### 3.5 DATS and DDP cooperated to increase endoplasmic reticulum stress in SGC-7901 cells

**FIGURE 5:**
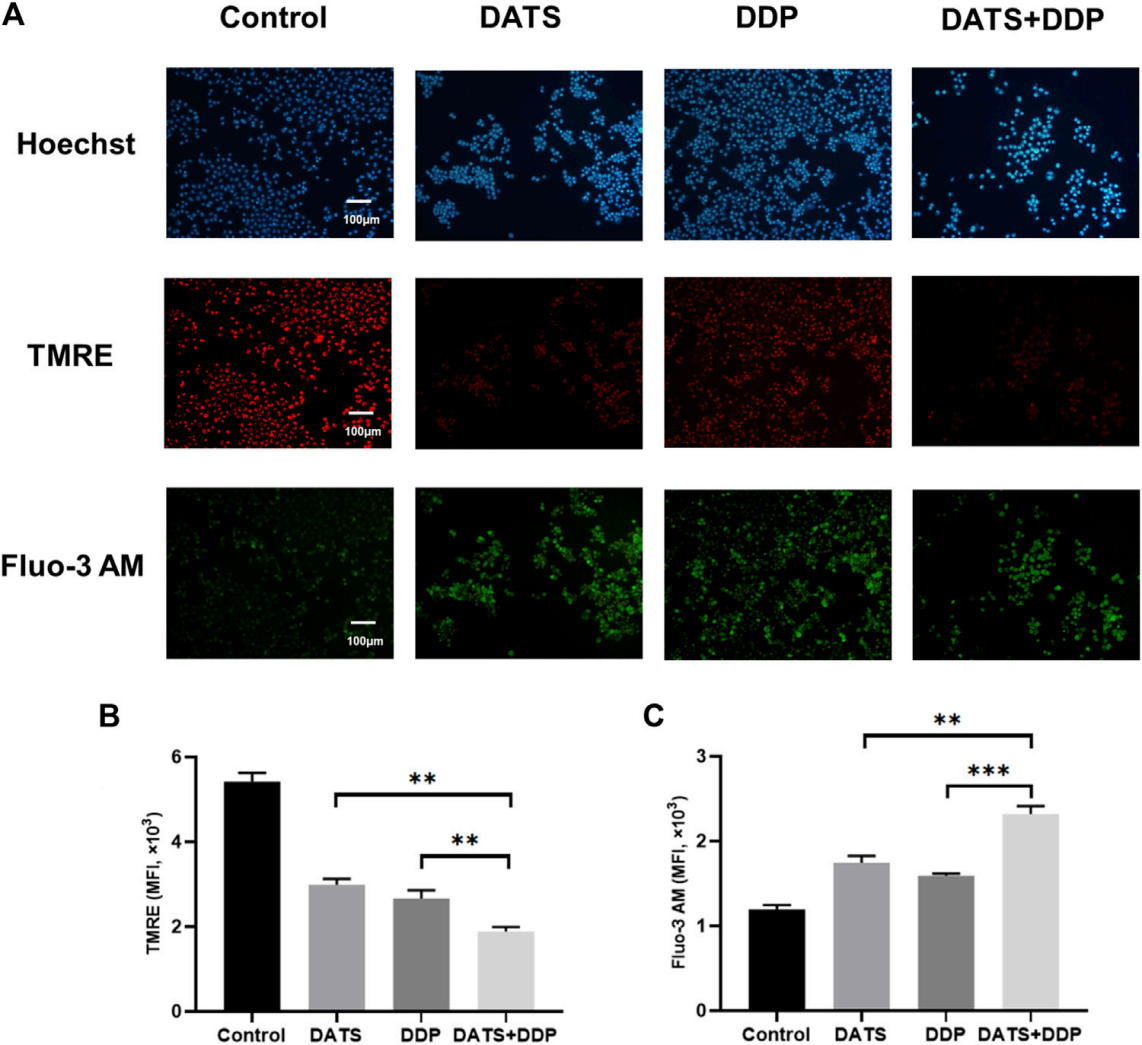
The effect of DATS and DDP on endoplasmic reticulum stress in SGC-7901 cells. **(A)** Representative immunofluorescence images of TMRE and Fluo-3 AM in SGC-7901 cells; **(B)** The expression of TMRE in SGC-7901 cells; **(C)** The expression of Fluo-3 AM in SGC-7901 cells. Data are shown as mean ± SD (*n* = 3). Compared with the DATS+DDP group, ^*^ *p* < 0.05, ^**^ *p* < 0.01, ^***^ *p* < 0.001.

Calcium ions are essential for the stability of the endoplasmic reticulum, and abnormal concentrations of calcium ions trigger endoplasmic reticulum stress, resulting in apoptosis. Changes in mitochondrial membrane potential are closely related to ER function of the endoplasmic reticulum. Therefore, the levels of calcium ions and mitochondrial membrane potential were evaluated to determine the effects of DATS+DDP on endoplasmic reticulum stress. As shown in Figure 5A, when DATS and DDP were used in combination, the mitochondrial membrane potential decreased significantly compared to both the DATS and DDP groups, according to the fluorescence intensity of TMRE by flow cytometry (^**^ *p* < 0.01, Figure 5B). Besides, the content of intracellular calcium ions (shown as fluorescence intensity of Fluo-3 a.m.) in the DATS+DDP group increased dramatically compared to that in the DATS and DDP groups (^**^ *p* < 0.01, ^***^ *p* < 0.001, respectively, Figure 5C). These results suggest that co-treatment with DATS and DDP triggers endoplasmic reticulum stress in SGC-7901 cells.

### 3.6 DATS and DDP regulated MAPK pathway, STAT3/PKC-δ and endoplasmic reticulum stress

**FIGURE 6:**
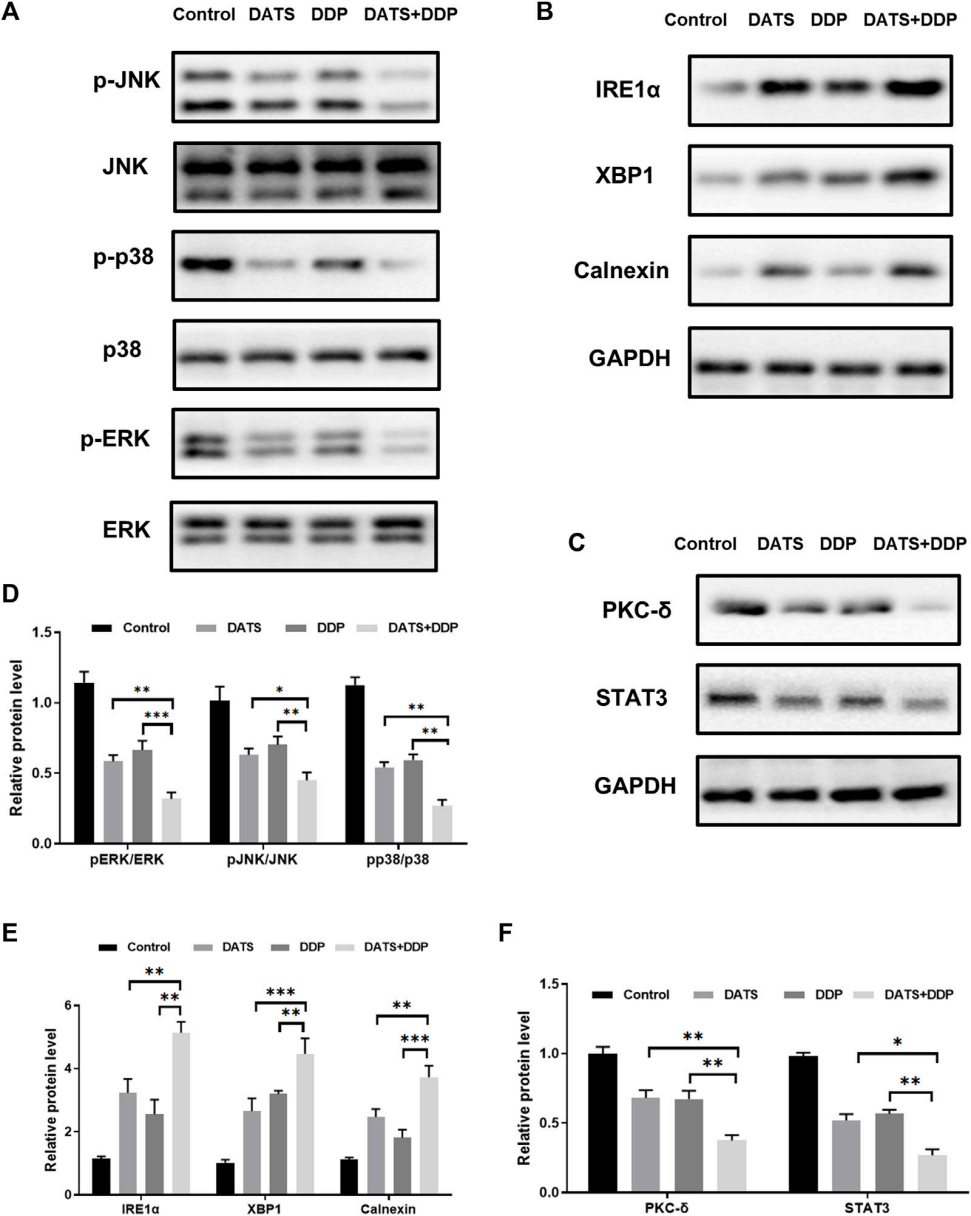
Effects of DATS and DDP on protein expressions of p-p38, p-ERK, p-JNK, STAT3, PKC-δ, IRE1α, XBP1 and Calnexin in SGC7901 cells. **(A)** p38, p-p38, ERK, p-ERK, JNK and p-JNK gel electrophoresis bands; **(B)** IRE1α, XBP1 and Calnexin gel electrophoresis bands; **(C)** STAT3 and PKC-δ gel electrophoresis bands; **(D)** p-p38, p-ERK and p-JNK protein expression; **(E)** IRE1α, XBP1 and Calnexin protein expression; **(F)** STAT3 and PKC-δ protein expression. Data are shown as mean ± SD (*n* = 3). Compared with the DATS and DDP group, ^*^ *p* < 0.05, ^**^ *p* < 0.01, ^***^ *p* < 0.001.

To verify the predictions of network pharmacology, the MAPK pathway, STAT3/PKC-δ, and endoplasmic reticulum stress were detected by Western blotting. The MAPK pathway was validated using p-p38, p-ERK, and p-JNK. STAT3/PKC-δ are closely related to the proliferation and invasion of GC cells. In addition, IRE1α, XBP1, and calnexin have been reported to be key proteins in endoplasmic reticulum stress generation (Wang et al., 2013). As important nodes for the combined effects of DATS and DDP, they have been explored using network pharmacology. Our results showed that the expression of the relevant proteins (p-p38, p-ERK, p-JNK, STAT3, and PKC-δ) was remarkably reduced after DDP or DATS treatment. Compared to the monotherapy groups, a considerable decrease in (p-p38, p-ERK, p-JNK, STAT3, and PKC-δ) was observed in the DDP and DATS groups (Figures 6A, C, D, F). IRE1α expression significantly increased after treatment with DDP+DATS (Figures 6B, E). Additionally, the levels of XBP1 and Calnexin showed a trend similar to that of IRE1α (Figures 6B, E).

### 3.7 DATS combined with DDP synergistically inhibited SGC-7901 tumor growth *in vivo*

**FIGURE 7:**
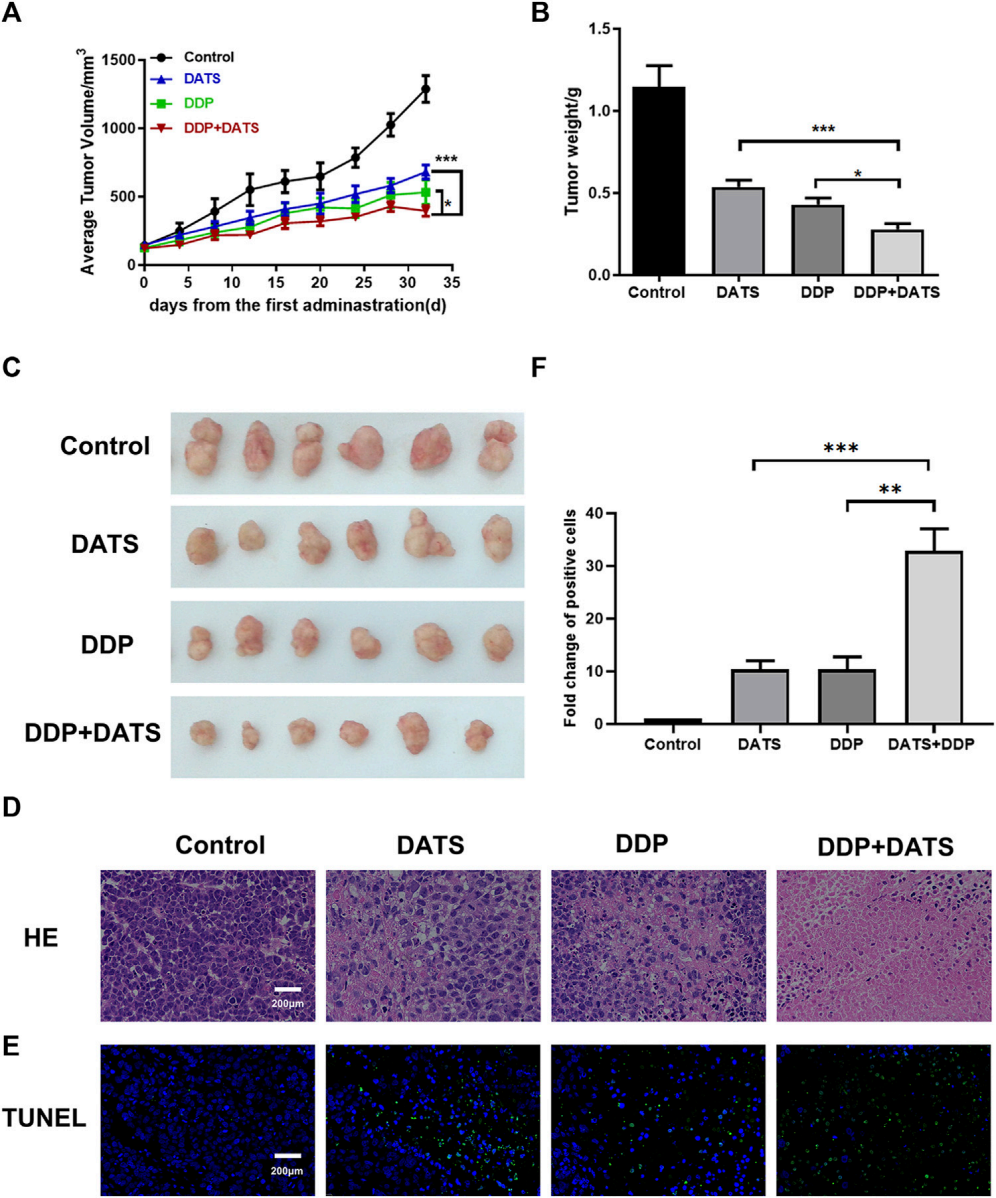
*In vivo* treatment of DATS and DDP in SGC-7901 tumor-bearing mice. **(A)** Average tumor volume; **(B)** Tumor weight; **(C)** Tumor morphology of mice; **(D)** HE staining results of tumor tissues; **(E)** TUNEL apoptosis results of tumor tissues; **(F)** Analysis of TUNEL-positive cells. Data are shown as mean ± SD (*n* = 6). Compared with the DATS+DDP group, ^*^ *p* < 0.05, ^**^ *p* < 0.01, ^***^ *p* < 0.001.

An SGC-7901 subcutaneous xenograft model in nude mice was established to further evaluate the *in vivo* effects of combined exposure. We observed that 30 mg/kg DATS and 4 mg/kg DDP effectively inhibited SGC-7901 xenograft after a 32-day treatment. In addition, the combined treatment showed a stronger inhibitory effect on TV and tumor weight (Figures 7A–C) than the DATS and DDP groups. Representative H&E-stained tumor sections demonstrated an increase in the area of cell-cell contact loss and tissue disintegration in the DATS, DDP, and the combined group, compared to the control group (Figure 7D), and notable necrosis and the fewest tumor cell infiltration were observed in DATS and DDP-treated tumor tissues. Compared to the control group, more TUNEL-positive cells were stained in the monotherapy groups. Additionally, the number of apoptotic tumor cells in the combination group was substantially higher than that in the DATS and DDP groups (^***^ *p* < 0.001 and ^**^ *p* < 0.01, respectively) (Figures 7E, F). Therefore, DATS synergistically enhanced the effect of DDP on tumor growth inhibition and apoptosis *in vivo*.

### 3.8 Combined treatment of DATS and DDP regulated MAPK pathway, STAT3/PKC-δ and endoplasmic reticulum stress *in vivo*

**FIGURE 8:**
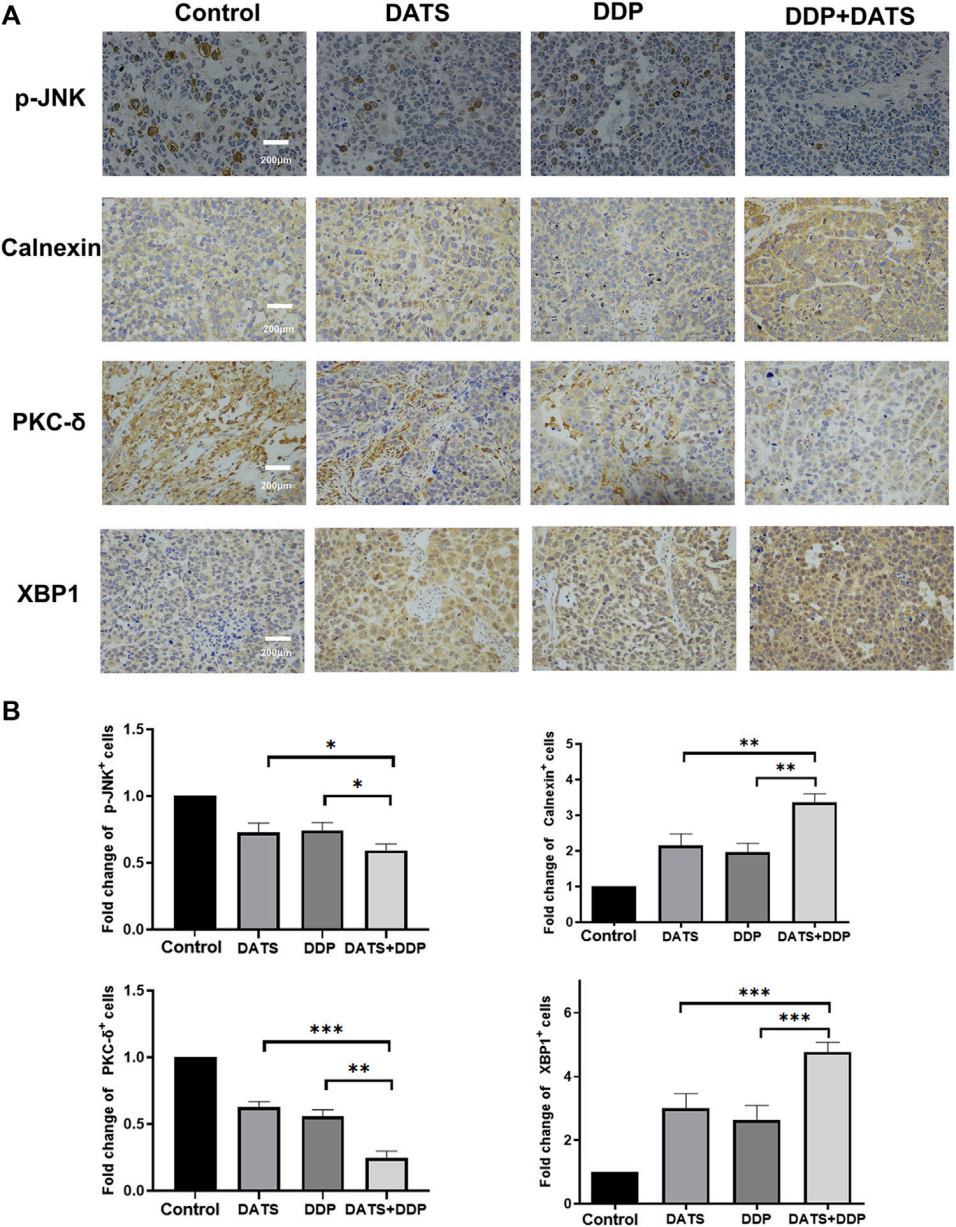
DATS and DDP regulated p-JNK, Calnexin, XBP1 and PKC-δ in SGC-7901 tumor-bearing mice. **(A)** Immunohistochemical detection of p-JNK, Calnexin, PKC-δ and XBP1 in tumor tissues; **(B)** Analysis of positive cells of p-JNK, Calnexin, PKC-δ and XBP1. Data are shown as mean ± SD (*n* = 3). Compared with the DATS+DDP group, ^*^ *p* < 0.05, ^**^ *p* < 0.01, ^***^ *p* < 0.001.

To verify the mechanism of the combination of DATS and DDP, the expression of p-JNK, Calnexin, XBP1 and PKC-δ was detected by immunohistochemistry. As shown in Figure 8, the expression of p-JNK and PKC-δ was decreased by DDP and DATS alone, while the expression of XBP1 and Calnexin, as signs of endoplasmic reticulum stress, was elevated compared to control group. In addition, the above changes were enhanced under the combined treatment of DATS and DDP compared with the groups treated with DATS or DDP alone.

## 4 Discussion

Garlic has been used in alternative medicine to treat a variety of diseases such as cardiovascular and cerebrovascular diseases (Sobenin et al., 2019), antigenic microbial infections, and several tumors (Nicastro et al., 2015; Mondal et al., 2022). DATS, the main component of garlic, was reported to exert anti-GC activity and enhance the chemosensitivity to GC of chemotherapeutic agent (Izdebska et al., 2016). DDP is the first-line drug used clinically for the treatment of GC, but its therapeutic effect is often affected by drug-resistance-associated problems (Fu et al., 2021). In addition, the use of high-dose DDP often causes strong side effects in patients (Dasari and Tchounwou, 2014). Therefore, by combining DATS with DDP, adverse effects may be improved by lowering the dose of DDP. On one hand, it is a promising way to reduce the development of tumor resistance by producing a synergistic anti-tumor effect. In our study, the synergistic therapeutic ability of DATS was found to enhance the anti-tumor effect of DDP in SGC-7901 cells and tumor-bearing models.

In terms of the molecular mechanism, the predictions of network pharmacology confirmed DATS played an important role in GC treatment combined with DDP in multiple pathways. PPI network analysis indicated combination of DATS and DDP may exert therapeutic effects on modulation of endoplasmic reticulum (ER) stress and STAT3/PKC-δ and MAPK signaling pathways, which was consistent with the KEGG pathway and GO analyses. The results offered preliminary insights into the mechanism of action of DATS combined with DDP against GC. Experimental mechanistic studies were performed to validate the protective effects of DATS combined with DDP against GC and its possible mechanism.

Mitogen activated protein kinase (MAPK) plays a critical role in progression of GC and drug resistance. Previous studies have suggested that activation in p38-MAPK pathway may be responsible for modulating multidrug resistance in the SGC-7901/VCR cell line (Guo et al., 2008). The role of garlic components in the MAPK pathway has also been partially reported (Chang et al., 2011). In addition, sustained activation of STAT3 maintains self-renewal of tumor stem cells, making tumors prone to recurrence, metastasis, and drug resistance (Aziz et al., 2007). Therefore, STAT3 plays an important role in tumor cell transformation, infiltration, metastasis, and progression. Moreover, STAT3 can continue to activate downstream PKC-δ and promote tumor survival in multiple ways. In our study, DATS combined with DDP synergistically enhanced cell growth inhibition, induction of cell apoptosis, inhibition of MAPK signaling phosphorylation, and STAT3/PKC-δ.

When cells are exposed to stress conditions such as nutrient deficiency, hypoxia, calcium imbalance, and oxidative stress, unfolded or misfolded proteins accumulate in the endoplasmic reticulum, resulting in ER stress (Oakes and Papa, 2015). If ER stress persists or is exacerbated, ER stress shifts from a pro-survival state to a pro-apoptotic state (Ferri and Kroemer, 2001). Therefore, promoting ER stress to initiate the apoptotic pathway is an anticancer therapeutic strategy (Yadav et al., 2014). In addition, persistent aggravation of ER stress also inhibits the MAPK pathway and STAT3 expression (Kim and Kim, 2018). In particular, studies focusing on cross-linking between natural products and existing chemotherapeutic agents via ER stress between apoptosis in cancer are of increasing interest (López-Antón et al., 2006; Law et al., 2010; Shi et al., 2016). It has been shown that certain drugs can induce apoptosis by inducing sustained activation of IRE1α-XBP1 and increased expression of calnexin thereby sustaining ER activation (Cubillos-Ruiz et al., 2015; Ryan et al., 2016; Wang et al., 2018). In our study, DATS combined with DDP synergistically enhanced the inhibition of ER stress.

In summary, the findings of our research confirmed that DATS increased the chemosensitivity to cisplatin in the treatment of GC, and its mechanism may be related to the MAPK pathway, STAT3/PKC-δ, and endoplasmic reticulum stress. These results suggest that DATS combined with DDP may be a new therapeutic strategy for the clinical treatment of GC. However, this study only focused on the synergistic anti-tumor activity and mechanism of DATS and DDP in normal gastric cancer cells, lacking relevant data on drug resistance and requiring further exploration.

## Data Availability

The raw data supporting the conclusions of this article will be made available by the authors, without undue reservation.
